# Disruption of Epithelial Barrier of Caco-2 Cell Monolayers by Excretory Secretory Products of *Trichinella spiralis* Might Be Related to Serine Protease

**DOI:** 10.3389/fmicb.2021.634185

**Published:** 2021-03-17

**Authors:** Chengyao Li, Xue Bai, Xiaolei Liu, Yuanyuan Zhang, Lei Liu, Lixiao Zhang, Fengyan Xu, Yong Yang, Mingyuan Liu

**Affiliations:** ^1^Key Laboratory of Zoonosis Research, Ministry of Education, College of Veterinary Medicine, Institute of Zoonosis, Jilin University, Changchun, China; ^2^Jiangsu Co-innovation Center for Prevention and Control of Important Animal Infectious Diseases and Zoonoses, Yangzhou, China

**Keywords:** *Trichinella spiralis*, excretory secretory products, serine protease, intestinal barrier, tight junction, invasion

## Abstract

The physical barrier is composed of epithelial cells which are joined together through intercellular connections. It serves to prevent pathogenic microorganisms from departing the intestinal lumen to invade the host. The excretory secretory (ES) products of *Trichinella spiralis* are critical for invasion. However, whether ES products of *T. spiralis* can act on the intestinal barrier is still unknown. In this study, the role of ES products of *T. spiralis* muscle larvae (*Ts*-ML-ES) in host invasion was studied by establishing an *in vitro* cell monolayers model. Barrier integrity analysis by a transmembrane resistance test and a paracellular permeability assay revealed that the *Ts*-ML-ES was able to destroy barrier function. It occurred *via* a reduction in the expression of tight junction (TJ) proteins, which was induced by serine protease. Furthermore, Western bolt analysis indicated that *Ts*-ML-ES reduced the expression of TJ proteins *via* the MAPK signaling pathway. Based on these data, we conclude that serine protease are likely the main factors from *Ts*-ML-ES that affect host intestinal barrier integrity by reducing the expression of TJs *via* the P38-MAPK signaling pathway. Serine protease in *Ts*-ML-ES might be a key invasion factor in *T. spiralis*.

## Introduction

For ingested pathogens such as bacteria and parasites, surviving encounters with the host stomach acid, proteases, mucus, and antimicrobial peptides (AMPs), they will encounter the intestinal epithelial cells (IECs) and the other intestinal barrier (Wood, 1993). The intestinal barrier defines the morphological and functional units responsible for protecting the intestinal mucosa, including intestinal flora, IECs and complex cytokine networks, AMPs, and many regulatory molecules (Meng et al., 2017). Epithelial cells and their connections constitute the physical barrier of the intestine, which serves a crucial function in preventing the invasion of pathogenic microorganisms. Epithelial cell connections consist of an apical junction complex of tight junctions (TJ), adhesion junctions, and desmosomes. The most significant limiting factor for intestinal permeability occurs at TJs (Capaldo and Nusrat, 2015). Several transmembrane proteins help to create TJs: TJ-related MARVEL (MAL and related proteins for vesicle trafficking and membrane) proteins (TAMP) including Occludin, tricellulin (limited to three-cell junction) and MarvelD3; claudins, a 27-member family, a single-span transmembrane protein that interacts in the same direction as the extracellular domain of claudins on adjacent cells; and, a single transmembrane immunoglobulin-like attachment adhesion molecule (JAMs; Odenwald and Turner, 2017). TJs play a key role in sealing intestinal tissue from the gut lumen (Oshima and Miwa, 2016). Disruption of TJs resulted in intestinal epithelial barrier dysfunction, and ultimately, increased microbial invasion of tissues (Betanzos et al., 2013).

*Trichinella spiralis* (*T. spiralis*) is a foodborne zoonotic nematode with an extensive host range. Hosts are infected by eating raw or undercooked meat that containing infective larvae (Murrell and Pozio, 2011; Yang et al., 2019). In the stomach by digestion of the meat, *T. spiralis* muscle larvae (ML) are released and activated by intestinal contents or bile after 0.9 h post-infection (hpi) to become intestinal infective larvae (IIL; Ren et al., 2013). The IIL interact with host IECs, invade the host intestinal epithelium, and develop into adult worms. The integrity of the intestinal epithelium barrier is important in protecting the host from infection by pathogens including *T. spiralis* infection. It was reported that when *T. spiralis* larvae invaded IECs, excretory secretory (ES) products remained in the host cell cytoplasm (Wang et al., 2012), implying that invasion of IECs by IIL might not simply depend on mechanical penetration to invade host cells, but also utilize ES products to assist invasion (Ren et al., 2011; Wang et al., 2017). Recent studies indicated that the ES products of *T. spiralis* contained some functional proteins, such as serine protease, serine protease inhibitor, and galectin, that might facilitate the invasion of intestinal epithelium by *T. spiralis* larvae (Song et al., 2018; Sun et al., 2018; Xu et al., 2018). In addition to interacting directly with epithelial cells, ES products from parasitic nematodes have also been reported to disrupt the integrity of the intestinal barrier. For example, the ES products of *Haemonchus contortus* and *Teladorsagia circumcincta* damaged intestinal epithelial barrier by altering expression of key TJ proteins (Rehman et al., 2016). However, the roles of ES products and other functional proteins of *T. spiralis* in the alterations of the intestinal epithelium barrier integrity are yet undefined.

In the present study, the effects of ES products of *T. spiralis* ML (*Ts*-ML-ES) on the integrity of Caco-2 monolayers were evaluated. We observed that *Ts*-ML-ES could disrupt the integrity of the intestinal barrier, change TJs, suggesting that ES products of *T. spiralis* play a role in assisting the invasion of the host, and which might be depends on serine protease.

## Materials and Methods

### Ethics Statement

All mice were handled strictly in accordance with the Animal Ethics Procedures and Guidelines of the People’s Republic of China. The protocol was approved by the Institutional Animal Care and Use Committee of Jilin University (Protocol # 20170318).

### Animals

All experimental animals were purchased from the YiSi Laboratory Animal Center (Changchun, China). The Wistar rats were specific-pathogen-free (SPF). Water and food were provided *ad libitum* during the entire experiment or up to 6 h prior to sacrifice, respectively.

### Parasite and ES Products Collection

*Trichinella spiralis* (ISS 534 strain) was maintained in Wistar rats, and ML were collected from the rat muscles at 42 day-post-infection (dpi) time point using a modified pepsin-hydrochloric acid digestion method. Collected ML were cultured in RPMI 1640 (Gibco, CA, United States) for 18 h at 37°C under 5% CO_2_ and 95% air (Li et al., 2010; Jiang et al., 2012).

Excretory secretory products of *T. spiralis* were performed as previously described (Bai et al., 2012). Briefly, 400 ml culture supernatants containing ES products were concentrated into 2 ml by centrifugation at 4°C using Ultra-15 3 K centrifugal filters (Millipore, MA, United States), and then resuspended in phosphate-buffered saline (PBS, PH 7.4). Protein concentrations were determined by using BCA Protein Assay (Bio-Rad, Hercules, CA, United States) and using BSA as the standard. The concentrated supernatants contained ES products, were referred to as *T. spiralis* ML (*Ts*-ML-ES) and were stored at −80°C.

To investigate the role of components within *Ts*-ML-ES, it was subjected to inactivation by heating in boiling water for 10 min. Also, *Ts*-ML-ES was pretreated with a protease inhibitor Phenylmethanesulfonyl fluoride (PMSF) for 10 min, at 1 μM working concentration.

### Cell Cultures

The human colorectal adenocarcinoma (Caco-2) cell line was maintained in 25-cm^2^ flasks containing Dulbecco’s modified Eagle’s medium (DMEM; Gibco) supplemented with 10% FBS (Gibco), 2-mM l-glutamine (Gibco), and 100 U/ml penicillin and 100 μg/ml streptomycin (Gibco) at 37°C in 5% CO_2_. For Transwell experiments, cells were seeded on 6-well (10^6^ cells/well) or 12-well (2 × 10^5^ cells/well) Transwell inserts (Corning, NY, United States) and grown to full confluency (21 days post-plating). The medium was changed every other day in week 1, and then daily in the subsequent 2 weeks.

### Cell Viability

The effect of *Ts*-ML-ES on the viability of host cells was assessed using a cell counting kit-8 (CCK-8) assay (Solarbio, Beijing, China). Cells were prepared to seed in a suspension of 10^5^ cells/100 μl in each well of a 96-well plate. The plates were pre-incubated for 24 h at 37°C under 5% CO_2_. Next, 10 μl *Ts*-ML-ES with different concentrations (0–50 μg/ml) were added to the culture plate. After incubation for 24 or 48 h, 10 μl CCK-8 solution was added to each well and was incubated for 1 h followed by the measurement of absorbance at 450 nm.

### Trans-Epithelial Electrical Resistance (TEER) Measurement

Trans-epithelial electrical resistance was used to assess the integrity of cell monolayers, and was measured using a Millicell-ERS volt-ohmmeter (Millipore) as previously described (Chen et al., 2019). Each well was measured three times and averaged as the resistance value of the well. Furthermore, each experiment also contained a cell-free blank well. Resistance value = (measured value-blank value) * film bottom area. Only the monolayers with epithelial resistance above 1,000 Ω•cm^2^ were used for subsequent experiments (Betanzos et al., 2013). Then cells were treated with *Ts*-ML-ES (25 μg/ml), TNF-α (20 ng/ml, Protech, United States), heat-inactivated *Ts*-ML-ES (25 μg/ml) or PMSF pretreated *Ts*-ML-ES (25 μg/ml) for 48 h. Notably, TNF-α was used as a positive control (Xiao et al., 2017), and medium was used as a negative control. For each experiment, three repetitions were analyzed for each group.

### Determination of Paracellular Permeability

Paracellular permeability of the Caco-2 monolayers grown on 12-well (2 × 10^5^ cells/well) was determined using Transwell inserts for 21 days. The cells were then apically treated with *Ts*-ML-ES (25 μg/ml), TNF-α (20 ng/ml), heat-inactivated *Ts*-ML-ES (25 μg/ml), or PMSF pretreated *Ts*-ML-ES (25 μg/ml) for 48 h. After discarding the supernatant, serum-free DMEM was added for 30 min at 37°C. The fluorescence intensity was measured as apical to basolateral flux of FITC-dextran (FD-4, M.W. 4 kDa, Sigma, United States) as previously described (O’Hara et al., 2012).

### qRT-PCR

Total RNA was extracted from Caco-2 cells using the Qiagen RNeasy kit (Qiagen, Germany). Reverse transcription of RNA was performed according to the instructions of TransScript One-Step gDNA Removal and cDNA Synthesis SuperMix (Trans, China). Next, the cDNA was amplified using the SYBR Green qRT-PCR Master Mix Kit (Roche, Switzerland). Human TJ protein genes, including Claudin-1, Occludin, and zonula occludens-1 (ZO-1) were amplified using specific primers, with expression being normalized to GAPDH. The experimental results were analyzed using the *Δ*Δct method. The primer sequences are shown in Table 1.

**Table 1:** Primer sequences and gene fragment size of amplified genes.

Gene name	Primer sequence(5'-3')	Product size
*GAPDH*	F: CTCCTCCTGTTCGACAGTCA	106 bp
	R: CGACCAAATCCGTTGACTCC	
*Claudin-1*	F: TGGTCAGGCTCTCTTCACTG	119 bp
	R: TTGGATAGGGCCTTGGTGTT	
*Occludin*	F: GGGCATTGCTCATCCTGAAG	85 bp
	R: GCCTGTAAGGAGGTGGACTT	
*ZO-1*	F: TTCACGCAGTTACGAGCAAG	141 bp
	R: TTGGTGTTTGAAGGCAGAGC	

### Western Blot

After 48 h of treatment, the M-per Mammalian Protein Extraction Reagent (Thermo Fisher Scientific, Waltham, MA, United States) was used to extract total protein according to the manufacturer’s instructions. Protein concentrations were measured using a BCA kit (Solarbio, Beijing, China). The proteins were resolved by SDS-PAGE, and subsequently transferred to PVDF membranes. After 1 h of 5% skim milk blocking, the primary antibodies, either anti-β-actin (1:3,000), anti-Claudin (1:2,000), anti-Occludin (1:2,000), anti-ZO-1(1:1,000), anti-MAPK (1:1,000), anti-pMAPK (1:1,000), anti-ERK1/2 (1:1,000), or anti-pERK1/2 (1:1,000), were incubated overnight at 4°C. The next day, the membrane was washed with TBST three times. Next, the HRP-goat-anti-rabbit antibody was incubated for 2 h at room temperature. After three washes, protein bands were revealed using ECL hypersensitive luminescent solutions (Thermo Fisher Scientific). All primary antibodies are rabbit monoclonal antibodies, and these are provided by Affinity (Affinity Biosciences, United States).

### Immunofluorescence Assay

After 21 days culture, the medium in the 12-well Transwell chamber was exhausted, and cells were washed three times with 500 μl PBS for 5 min each time. Then 500 μl 4% paraformaldehyde was added to each chamber, and the cells were fixed on ice for 20 min. After permeabilization for 15–20 min with 0.5% Triton X-100 (in PBS), cells were blocked with 2% BSA in PBS for 1 h at room temperature. Claudin-1, Occludin, and ZO-1 primary antibody solutions were prepared at concentrations of 1:200, 1:100, and 1:100, respectively. To each chamber, 100 μl antibody was added and incubated overnight at 4°C. Secondary antibody (FITC-goat anti-rabbit; Abcam, United Kingdom) diluted 1:1,000 in PBS was incubated for 1 h at room temperature. Note that it is strictly protected from light from this step. Subsequently, DAPI (Invitrogen) was used for counterstaining of the nucleus. Finally, the membrane was placed on a glass slide with anti-fluorescence quencher (Invitrogen) and observed using a laser scanning confocal microscopy (Fluoview FV 1000, Japan). All primary antibodies are rabbit monoclonal antibodies, which were provided by Affinity (Affinity Biosciences).

### Transmission Electron Microscopy

Caco-2 cells were inoculated on 6-well Transwell for about 21 days. The culture medium was removed and the cells were washed with PBS. The cells were gently scraped off the microporous membrane and placed in 2.5% glutaraldehyde. The cells were shaken to disperse, fixed for 3 min, and pelleted by centrifugation. Next, 2.5% stationary liquid was added, and the samples were incubated at 4°C. The sample was handed over to the director of the Instrument Center. After embedding and sectioning, the ultrastructure, TJs, and microvilli of the cells were observed by transmission electron microscopy (Hitachi Limited, Japan).

### Statistical Analysis

All statistical analyses of the data were performed using GraphPad Prism 8 (GraphPad). The TEER and fluorescence data were expressed as the mean value ± standard deviation, and the intra‐ and intergroup differences were analyzed using one-way ANOVA or Student’s *t*-test.

## Results

### The ES Products of *T. spiralis* Destroy the Integrity of the Intestinal Barrier

**Figure 1:**
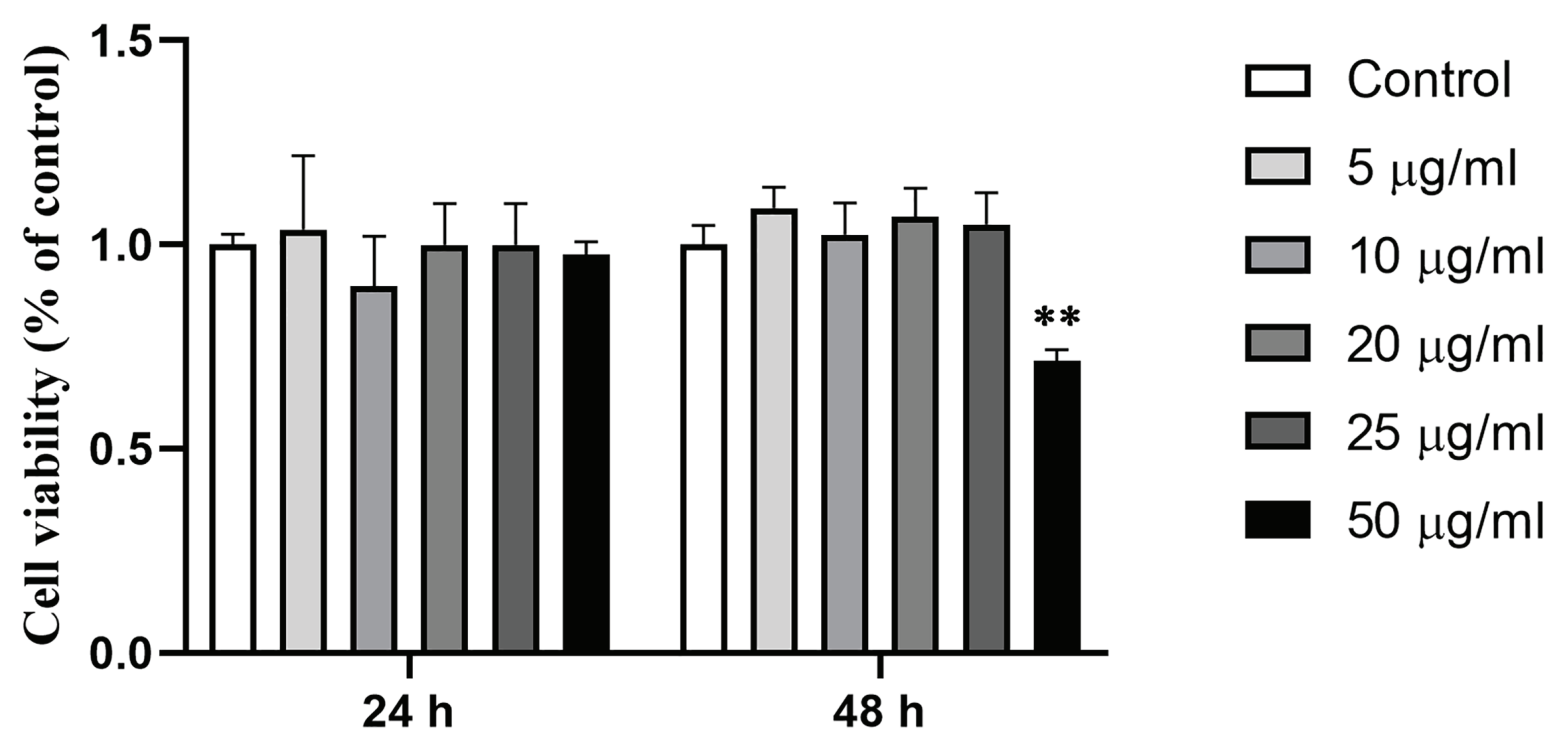
Effect of *Ts*-ML-ES on Caco-2 cell viability. Viability of the cell monolayers were determined by CCK-8 assay after treatment with 0 (negative control), 5, 10, 20, 25, or 50 μg/ml *Ts*-ML-ES for 24 and 48 h, respectively. Values are presented as mean ± SD, *n* = 3. ∗∗*p* < 0.01 by one-way ANOVA with Tukey’s posttest.

When Caco-2 cells were treated with *Ts*-ML-ES at 0–50 μg/ml for 24 or 48 h, significant reduction of viability was observed only in the 50 μg/ml × 48 h treatment group, but not in any of other groups (Figure 1). 25 μg/ml *Ts*-ML-ES were used in subsequent experiments.

**Figure 2:**
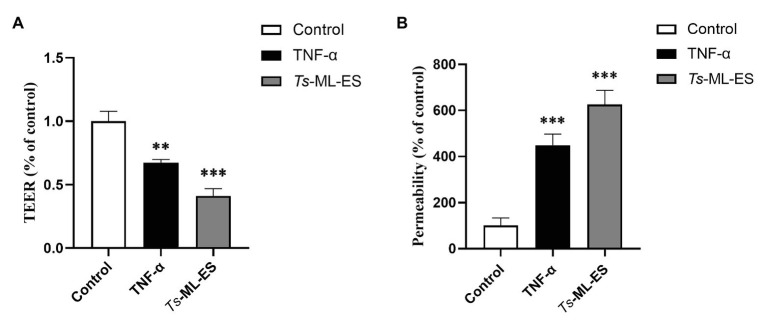
The *Ts*-ML-ES destroys the barrier integrity of Caco-2 cell monolayers. **(A)** *Ts*-ML-ES decreases trans-epithelial electrical resistance (TEER) across the Caco-2 monolayers. **(B)** *Ts*-ML-ES increases paracellular permeability of FITC-dextran in Caco-2 cell monolayers. The apical-to-basolateral flux of FITC-dextran across Caco-2 monolayers cultured in the Transwell system was monitored for 1 h after the addition of dextran in the absence (control) and presence of *Ts*-ML-ES or TNF-α. Data represent the mean ± SD from three independent experiments. ∗∗*p* < 0.01, ∗∗∗*p* < 0.001 by one-way ANOVA with Tukey’s posttest.

In TEER, the electrical resistance membranes increased over time and stabilized after 20 days reaching 1,400 Ω (Supplementary Figure S1). Upon treatment by *Ts*-ML-ES and TNF-α (a positive control), the electrical resistance decreased by 40 and 60%, respectively (Figure 2A). In paracellular permeability assay, the FITC-dextran across the membranes increased by 300 and 500% in groups treated with *Ts*-ML-ES, a TNF-α, respectively (Figure 2B). These observations were indications of a loss of Caco-2 cell junctions.

According to ToxinSensor™ Chromogenic LAL Endotoxin Assay Kit (Genscript, Piscataway, NJ, USA), the *Ts*-ML-ES solution at 25 μg/ml contained 0.2 EU endotoxin, which was equivalent to 0.02 ng/ml of LPS (lipopolysaccharide) based on the standard of the United States Pharmacopeia. Previous literature report showed that trace amounts of LPS could not reduce TEER (He et al., 2020). The results showed that the profile of changes in TEER induced by the *Ts*-ML-ES was not related to the LPS trace.

### The ES Products of *T. spiralis* Disrupt Intestinal Barrier by Affecting Tight Junction Proteins

**Figure 3:**
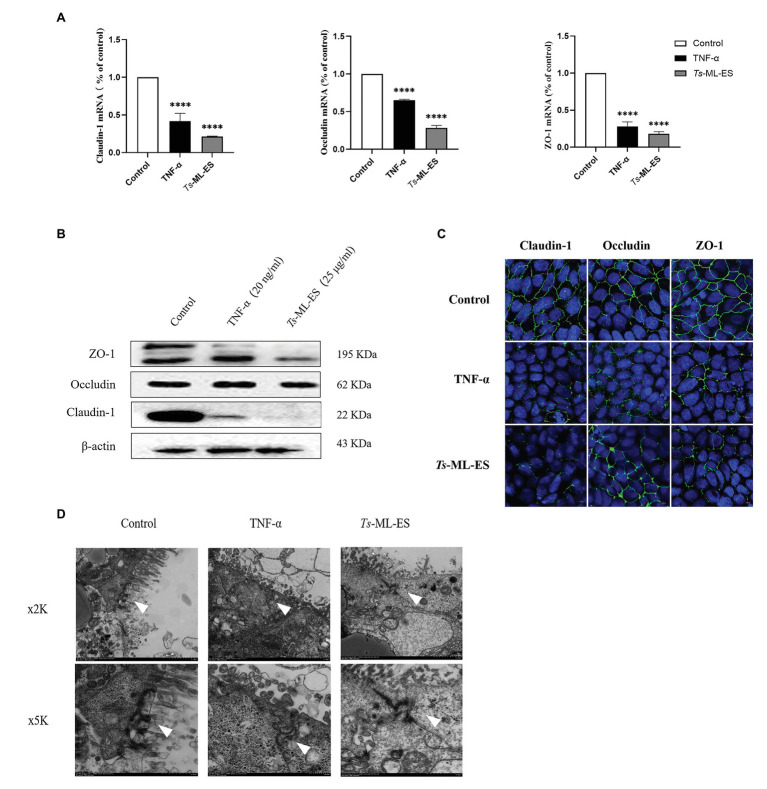
*Ts*-ML-ES decreases the expression of tight junction (TJ) proteins. **(A)** Expression of claudin-1, Occludin, and ZO-1 mRNA are altered in Caco-2 cells 48 h after treatment with *Ts*-ML-ES. mRNAs expression of stimulated cells (normalized with GAPDH) relative to unstimulated cells as mean ± SD from three independent experiments. ∗∗∗∗*p* < 0.0001 by one-way ANOVA with Tukey’s posttest. **(B)** The effect of *Ts*-ML-ES on the expression of Claudin-1, Occludin, and ZO-1 in Caco-2 cells. Fully differentiated Caco-2 cells were incubated with 25 μg/ml *Ts*-ML-ES (48 h). Cells without any treatments are designated as the negative control (NC) and those treated with TNF-α as the positive control (PC). β-actin was used as the internal control. Representative blots of three independent experiments are shown. **(C)** Effect of apical membrane exposure in control, TNF-α, or *Ts*-ML-ES treatment of *Ts* on the location of Claudin-1, Occludin, or ZO-1 in the TJs of Caco-2 cell monolayers. The merged images demonstrate the three TJ proteins in green. DNA was stained with DAPI (blue) to reveal the positions of the nuclei. **(D)** Transmission electron microscopy (TME) of Caco-2 cells incubated for 48 h with 20 ng/ml TNF-α, and 25 μg/ml *Ts*-ML-ES. The cells were then fixed with glutaraldehyde in preparation for TEM imaging. Arrowheads: Structure of TJ protein.

To verify whether intestinal barrier disruption is associated with TJ protein expression, changes in TJ gene and protein expression were examined. Results obtained from qPCR indicated decreased gene expression of the three TJ protein genes in response to *Ts*-ML-ES treatment (Figure 3A). Furthermore, the amount of TJ protein expression also decreased as demonstrated by Western blot. It is worth noting that the expression of Claudin-1 was markedly reduced, but there was no significant change in expression of Occludin (Figure 3B). Therefore, we have reason to suspect that *Ts*-ML-ES reduced the expression of TJ proteins mainly through interaction with Claudin-1. To support these observations, immunofluorescence assay (IFA) was performed. Not only could IFA show the subcellular localization of the three proteins, but it also provided supporting data for the q PCR and WB results as well. Specifically, *Ts*-ML-ES does attenuate the expression of TJ proteins between cells (Figure 3C). Finally, to locate the TJ proteins between cells, transmission electron microscopy (TEM) was used. In the untreated group, the cells were well-grown and the microvilli were neatly arranged. The TJs were located at the top of the outer membrane, showing a dense band structure. In the group treated with *Ts*-ML-ES, the TJs were relaxed, the intercellular space was widened, and the microvilli were arranged sparsely and irregularly. In group treated with TNF-α, ultra-structural changes were not particularly significant, although some mild pathology was observed, relative to that of the control group (Figure 3D).

### Serine Protease in ES Products of *T. spiralis* Compromise of Intestinal Barrier Integrity

**Figure 4:**
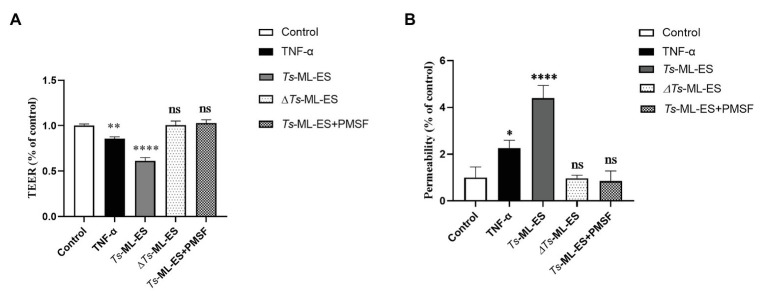
Inhibition of serine protease activity in *Ts*-ML-ES reduced barrier disruption. **(A)** The TEER of Caco-2 cells incubated with TNF-α (20 ng/ml), *Ts*-ML-ES (25 μg/ml), Heat-inactivated *Ts*-ML-ES (25 μg/ml) or PMSF pretreated *Ts*-ML-ES (25 μg/ml). **(B)** The paracellular permeability of FITC-dextran in Caco-2 cell monolayers. Caco-2 cells were incubated with the same stimuli as above. Data represent the mean ± SD of the mean of three independent experiments. ∗*p* < 0.05, ∗∗*p* < 0.01, ∗∗∗∗*p* < 0.0001 by one-way ANOVA with Tukey’s posttest.

**Figure 5:**
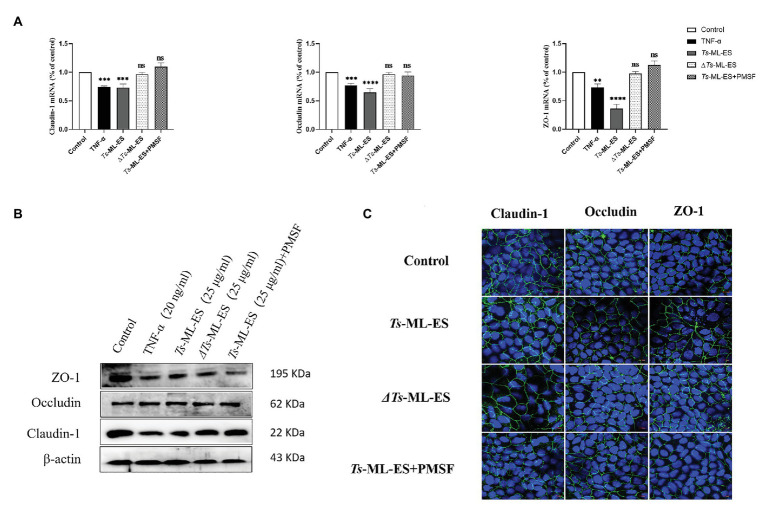
Serine protease affects the expression of TJ proteins. The Caco-2 cells were treated with TNF-α (20 ng/ml), *Ts*-ML-ES (25 μg/ml), heat-inactivated *Ts*-ML-ES (25 μg/ml), or PMSF pretreated *Ts*-ML-ES (25 μg/ml). **(A)** Expression of Claudin-1, Occludin, and ZO-1 mRNA. Data represent the mean ± SD from the mean of three independent experiments. ∗∗*p* < 0.01, ∗∗∗*p* < 0.001, ∗∗∗∗*p* < 0.0001 by one-way ANOVA with Tukey’s posttest. **(B)** Protein expression of Claudin-1, Occludin, and ZO-1. β-actin was used as the internal control. Representative blots of three independent experiments are shown. **(C)** IFA of Claudin-1, Occludin, and ZO-1. The merged images represent the three TJ proteins in green. DNA was stained with DAPI (blue) to reveal the positions of the nuclei.

To investigate the functional component of *Ts*-ML-ES that disrupts the intestinal barrier. Heat-inactivated *Ts*-ML-ES was used to treat Caco-2 cells. In another group, PMSF, a protease inhibitor that primarily inhibits serine protease, was added to pretreat the *Ts*-ML-ES for 10 min (Vandana et al., 2018). Cell barrier integrity tests showed that neither heat-inactivated *Ts*-ML-ES nor inhibitor-pretreated *Ts*-ML-ES could decrease resistance and increase cell permeability (Figure 4). However, the addition of PMSF alone did not affect the observed resistance (Supplementary Figure S2). As for the expression of TJ protein genes, Claudin-1 and ZO-1 expression were significantly decreased following exposure to active *Ts*-ML-ES only, but this effect was not achieved by inactivated *Ts*-ML-ES or inhibitor-pretreated *Ts*-ML-ES (Figure 5A). In addition, Western blot and IFA results corroborated the observations that inactivated *Ts*-ML-ES or inhibitor-pretreatment failed to reduce the expression of TJ proteins (Figures 5B,C). These results suggested that the active serine protease in *Ts*-ML-ES played an important role in compromising intestinal barrier integrity.

### The ES Products of *T. spiralis* Reduce the Expression of Tight Junction Proteins *via* the MAPK Signaling Pathway

**Figure 6:**
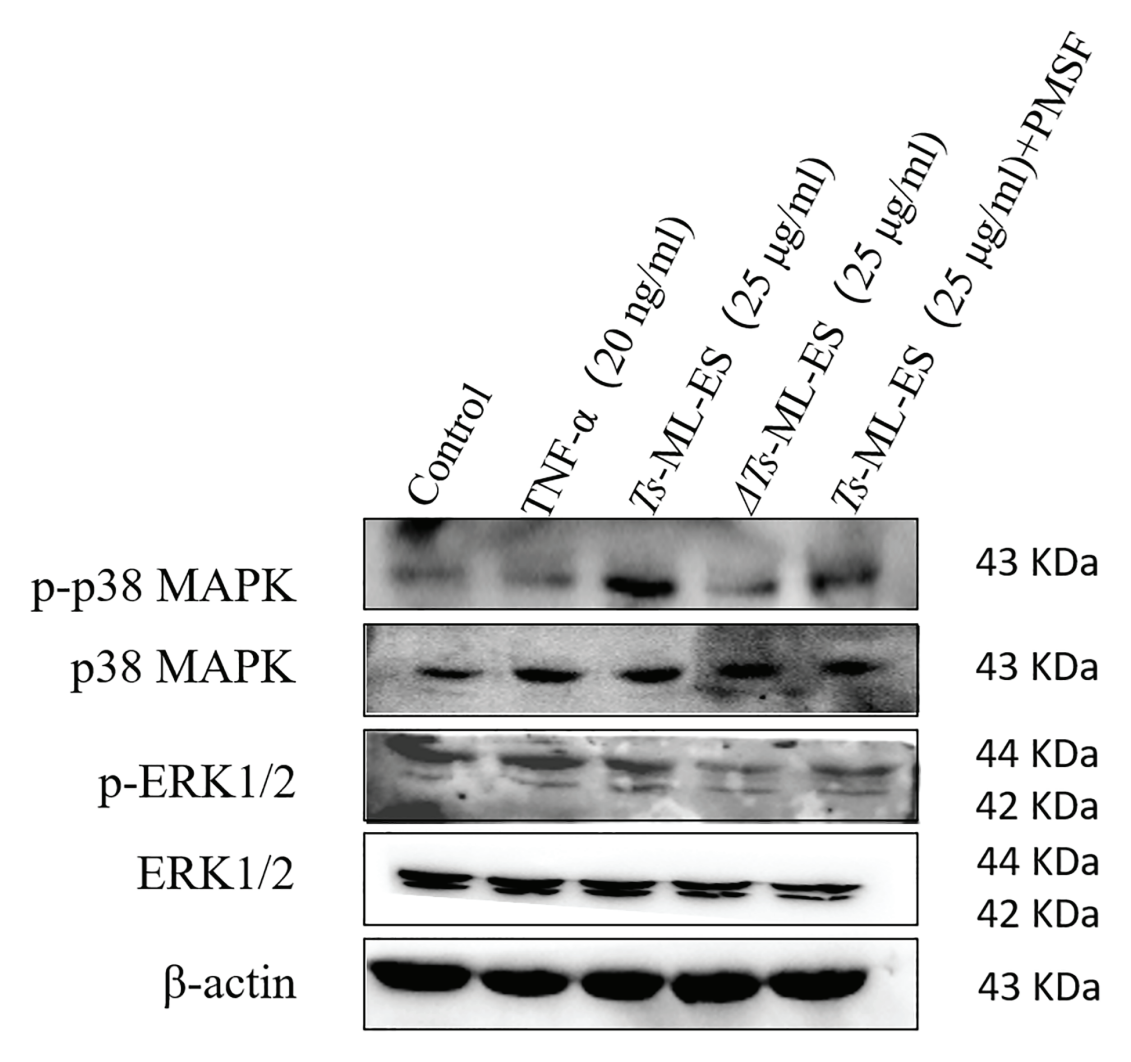
The effect of *Ts*-ML-ES on MAPK expression. The activation of ERK1/2 and P38 in *Ts*-ML-ES-treated cells. Caco-2 cells were incubated with TNF-α (20 ng/ml), *Ts*-ML-ES (25 μg/ml), heat-inactivated *Ts*-ML-ES (25 μg/ml), or PMSF-*Ts*-ML-ES (25 μg/ml). β-actin was used as the internal control. Representative blots of three independent experiments are shown.

In order to further understand the mechanism of *Ts*-ML-ES compromising TJ proteins, the mitogen-activated protein kinase (MAPK) signaling pathway was analyzed. As is shown in Figure 6, phosphorylation of p38 MAPK increased significantly following *Ts*-ML-ES treatment. No significant change in the levels of phosphorylated p38 in samples treated with heat-inactivated *Ts*-ML-ES or inhibitor-pretreated *Ts*-ML-ES. In addition, there was no significant effect on ERK1/2 and phospho-ERK1/2 within the MAPK pathway. These results indicated that the ES products of *T. spiralis* reduced the expression of TJ proteins *via* the p38-MAPK signaling pathway.

## Discussion

The intestinal epithelial barrier is vital in the prevention of pathogenic microorganism invasion, as well as limiting the access of pathogenic antigens and toxic substances into the systemic circulation (Wang et al., 2016). The destruction of the intestinal barrier can cause these substances to enter the circulatory system and adversely affect the body (Chen et al., 2019). The epithelial barrier is usually reflected by TEER and paracellular permeability (Srinivasan et al., 2015). When the barrier is broken, a decrease in TEER and an increase in cellular permeability are observed. Some intestinal parasites, especially protozoa, cause damage to the intestinal barrier, such as *Cryptosporidium*, *Toxoplasma*, and *Giardia* (Briceno et al., 2016; Kumar et al., 2018; Ma’ayeh et al., 2018). However, intestinal worms often damage the intestinal barrier through mechanical movement (Ren et al., 2011). In addition, worms may also rely on the released ES products to affect the intestinal barrier. It has been reported that ES products of *Trichuris suis* reduced barrier function, suppressed LPS-inducing cytokine production, and increased the production of Th2 response-inducing cytokines (Hiemstra et al., 2014). Similar conclusions were reported for *H. contortus*, which confirmed the ability of ES products of adult *H. contortus* and *T. circumcincta* to disrupt TJs of cultured epithelial cells (Rehman et al., 2016). However, ES products of *T. spiralis* mediated destruction of the intestinal barrier has not been reported. In this study, the effect of ES products of *T. spiralis* on the intestinal barrier were characterized. It was observed that *Ts*-ML-ES could decrease the TEER of Caco-2 cell monolayers and increase the paracellular permeability, which is a signal of barrier destruction. Therefore, it may play an important role in tissue damage leading to *T. spiralis* infective larvae invasion.

Many reports have shown that TJ proteins are mechanistic targets of parasite invasion and infection (Hiemstra et al., 2014; Briceno et al., 2016). TJs act as a semi-permeable barrier for the transport of ions, solutes, and water (Bonazzi and Cossart, 2011). The TJ complex is mainly composed of claudins, occludin, and Zos (Furuse et al., 1993). The claudin family consists of at least 24 proteins, of which differential expression characteristics determine tissue-specific changes in epithelial resistance and paracellular permeability (Gunzel and Yu, 2013). Claudin-1 is widely expressed in the intestinal epithelium, and its differential expression and characteristics determine tissue-specific changes in epithelial resistance and paracellular permeability. It has been proposed to an important component of the TJ structure (Garcia-Hernandez et al., 2017). Occludin, another TJ protein, has also been shown to regulate cell permeability by acting on claudins. However, some studies have put forward a controversial hypothesis that occludin may be a regulator of TJ, rather than an integral component. This is based on studies in occludin-defective mice, where the intestinal barrier is intact (Schulzke et al., 2005; France and Turner, 2017). This controversy is supported by the Western blot results presented here. As for ZO-1, it has the same efficacy as Claudin-1, and when the parasite invades, a decrease in expression was observed. Many studies have reported that different foreign substances can cause the destruction of the intestinal barrier by targeting different TJ proteins (Furuse et al., 1994; Betanzos et al., 2013). In this experiment, changes in expression of three TJ proteins were examined. The gene and protein expression levels of Claudin-1 and ZO-1decreased to varying degrees upon exposure to *Ts*-ML-ES. In particular, the expression of Claudin-1 protein was the most obvious. Although the expression of occludin mRNA decreased, the protein level was not significantly affected. The non-linear relationship between the mRNA and protein may be related to post-transcription efficiency and degradation rate. Further studies are needed to examine this hypothesis. Collectively, the data presented here show that *Ts*-ML-ES mainly acts on Claudin-1 to compromise the integrity of the intestinal barrier.

The components in *Ts*-ML-ES are quite complex, and it is not known what is responsible for its effects. To answer this question, *Ts*-ML-ES was inactivated. It was observed that inactivated *Ts*-ML-ES did not affect barrier function and TJs, suggesting that active protein in *Ts*-ML-ES plays an important role. Serine proteinases with chymotrypsin, elastase, or trypsin-like activities are the most abundant proteinases of ES products or crude extract proteins from *T. spiralis* (Hu et al., 2020). Antibodies induced by serine proteinases of adult *T. spiralis* and larvae could inhibit their enzymatic activity and may participate in reducing tissue damage during *Trichinella* invasion and migration (Yong et al., 2015). During *T. spiralis* invasion of epithelial cells, the larvae are able to release a variety of glycoproteins, which are high in glycoantigens and Tyvelose. Monoclonal antibodies against Tyvelose can prevent infection, which predicts the important role of Tyvelose-associated glycoproteins in IEC invasion. However, these glycoproteins were verified to be serine proteases (Romaris et al., 2002). Furthermore, r*Ts*Serp has been reported to help larvae invade IECs during infection (Yue et al., 2020). Therefore, it is likely that serine proteases act on TJ proteins to achieve the intestinal barrier disruption. In order to determine whether serine protease is the functional unit of *Ts*-ML-ES that destroys the intestinal barrier, the serine protease inhibitor was used. PMSF acts on the active site of the sulfonated serine residue of serine protease to thereby inhibit it. *Ts*-ML-ES pretreated with PMSF yielded the same results as was observed with heated-inactivated *Ts*-ML-ES, implying that a significant part of activity is provided by the serine protease. It is well known that proteases can act as signal molecules by activating protease-activated receptor (PAR) family members (PAR-1, -2, -3, and -4). PAR-2 has been reported to be activated by the protease of the parasite and is closely related to the regulation of inflammation, permeability, and ion transport (Adams et al., 2011; Fernandez-Blanco et al., 2013). Future experiments are needed to investigate the roles of serine protease in barrier disruption, including the use of recombinantly expressed proteins and other types of serine protease inhibitors. In addition, it is necessary to further explore whether the key component of ES, serine protease, can activate downstream pathways through PAR-2 to cause the destruction of the intestinal barrier.

The MAPK signaling pathway is a well-known signal transduction pathway that regulates a variety of cellular activities (Peyssonnaux and Eychene, 2001). Importantly, the MAPK pathway plays a critical role in the regulation of intestinal epithelial barrier functions (Koopmans et al., 2011). Studies have shown that some substance can cause the intestinal barrier to be destroyed, thereby increasing the expression of MAPK (Böhringer et al., 2016; Gao et al., 2017). Cvetkovic et al. (2016) once reported that *Ts*-ML-ES can increase the phosphorylation of ERK and P38 in DC cells. Therefore, in this experiment, the expression of MAPK related molecules was examined in Caco-2. It was observed that only the active *Ts*-ML-ES increased the expression of phosphorylated P38. Neither the heated-inactivated protein nor the inhibitor-pretreated *Ts*-ML-ES altered expression. Furthermore, expression of the ERK-related molecules ERK1/2 and p-ERK1/2 did not change significantly. Since the activation of ERK1/2 is mostly transient, the cells were processed for 48 h in our study. Thus, it was concluded that the serine protease of *Ts*-ML-ES is a likely regulator of TJ proteins through the P38-MAPK pathway.

The intestinal barrier consists of four layers. In addition to the physical barriers mentioned in this article, there are microbial, chemical, and immune barriers. Microbial barrier, as well as the role that intestinal flora plays in the interaction between the host and *T. spiralis* is an important focus for the future research. In addition, after the intestinal barrier is damaged, the role of the host immune cells and molecules in barrier repair and combating *T. spiralis* in the host is also of particular interests.

## Conclusion

The study showed that *Ts*-ML-ES at 25 μg/ml disrupts the Caco-2 cell monolayer barrier function by decreasing the expression of TJ proteins, mainly the down-regulation of Claudin-1. An active serine protease component in the *Ts*-ML-ES was necessary to produce the effect, suggesting that serine protease might play an important role in the *T. spiralis* invasion of the intestinal barrier.

## Data Availability Statement

The original contributions presented in the study are included in the article/Supplementary Material, further inquiries can be directed to the corresponding authors.

## Ethics Statement

The animal study was reviewed and approved by Institutional Animal Care and Use Committee of Jilin University.

## Author Contributions

The study was conceived and designed by YY and ML. CL performed the experiments. XB, XL, and CL analyzed the data. CL and XB wrote the manuscript. YZ and LL provided experimental technical support. LZ, FX, YY, and ML improved the manuscript. All authors contributed to the article and approved the submitted version.

### Conflict of Interest

The authors declare that the research was conducted in the absence of any commercial or financial relationships that could be construed as a potential conflict of interest.
