# Supplementary material for: Disruption of Epithelial Barrier of Caco-2 Cell Monolayers by Excretory Secretory Products of Trichinella spiralis Might Be Related to Serine Protease
Source: Front Microbiol. 2021 Mar 17;12:634185. doi: 10.3389/fmicb.2021.634185 (PMC8013981; doi:10.3389/fmicb.2021.634185)
Supplement: Supplementary file 1 [file Data_Sheet_1.pdf]

Supporting information

S1 Appendix.

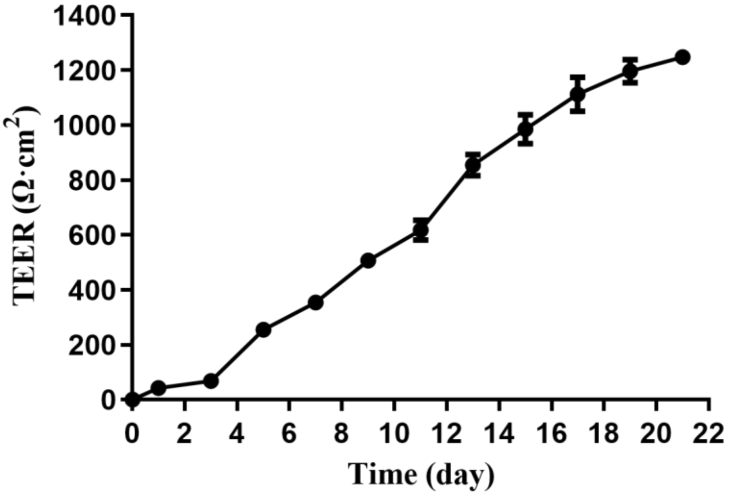

S1 Appendix. Changes in resistance of Caco-2 cells after 21 days of culture. Values are presented as mean ± SD, n = 3.

S2 Appendix.

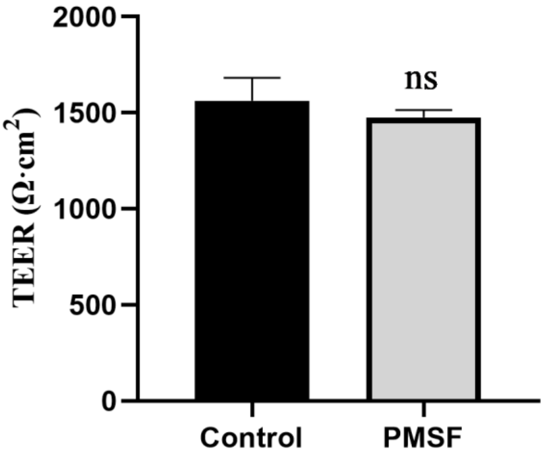

S2 Appendix. The effect of PMSF on TEER. Values are presented as mean ± SD, n = 3. NS=No significant (t-tests).
